# Improved residue contact prediction using support vector machines and a large feature set

**DOI:** 10.1186/1471-2105-8-113

**Published:** 2007-04-02

**Authors:** Jianlin Cheng, Pierre Baldi

**Affiliations:** 1 School of Electrical Engineering and Computer Science, University of Central Florida, Orlando, FL 32816-2362, USA; 2 School of Information and Computer Sciences, University of California Irvine, Irvine, CA 92617, USA

## Abstract

**Background:**

Predicting protein residue-residue contacts is an important 2D prediction task. It is useful for *ab initio *structure prediction and understanding protein folding. In spite of steady progress over the past decade, contact prediction remains still largely unsolved.

**Results:**

Here we develop a new contact map predictor (SVMcon) that uses support vector machines to predict medium- and long-range contacts. SVMcon integrates profiles, secondary structure, relative solvent accessibility, contact potentials, and other useful features. On the same test data set, SVMcon's accuracy is 4% higher than the latest version of the CMAPpro contact map predictor. SVMcon recently participated in the seventh edition of the Critical Assessment of Techniques for Protein Structure Prediction (CASP7) experiment and was evaluated along with seven other contact map predictors. SVMcon was ranked as one of the top predictors, yielding the second best coverage and accuracy for contacts with sequence separation >= 12 on 13 *de novo *domains.

**Conclusion:**

We describe SVMcon, a new contact map predictor that uses SVMs and a large set of informative features. SVMcon yields good performance on medium- to long-range contact predictions and can be modularly incorporated into a structure prediction pipeline.

## Background

Predicting protein inter-residue contacts is an important 2D structure prediction problem [1]. Contact prediction can help improve analogous fold recognition [2,3] and *ab initio *3D structure prediction [4]. Several algorithms for reconstructing 3D structure from contacts have been developed in both the structure prediction and determination (NMR) literature [5-8]. Contact map prediction is also useful for inferring protein folding rates and pathways [9,10].

Due to its importance, contact prediction has received considerable attention over the last decade. For instance, contact prediction methods have been evaluated in the fifth, sixth, and seventh editions of the Critical Assessment of Techniques for Protein Structure Prediction (CASP) experiment [11-15]. A number of different methods for predicting contacts have been developed. These methods can be classified roughly into two non-exclusive categories: (1) statistical correlated mutations approaches [16-22]; and (2) machine learning approaches [23-34]. The former uses correlated mutations of residues to predict contacts. The latter uses machine learning methods such as neural networks, self organizing map, hidden Markov models, and support vector machines to predict 2D contacts from the primary sequence, as well as other 1D features such as relative solvent accessibility and secondary structure.

In spite of steady progress, contact map prediction remains however a largely unsolved challenge. Here we describe a method that uses support vector machines together with a large set of informative features to improve contact map prediction. On the same data set, SVMcon outperforms the latest version of the CMAPpro contact map predictor [28,35] and is ranked as one of the top predictors in the blind and independent CASP7 experiment.

## Results and Discussion

We first compare SVMcon with the latest version of CMAPpro on the same benchmark dataset. Then we describe the performance of SVMcon along with several other predictors during the CASP7 experiment.

### Comparison with CMAPpro on the same Benchmark

SVMcon is trained to predict medium- to long-range contacts (sequence separation >= 6) as in [36], which are not captured by local secondary structure. We train SVMcon on the same dataset used to train CMAPpro [28,35] and test both programs on the same test dataset. The training dataset contains 485 proteins and the test dataset contains 48 proteins. The sequence identity between the training and testing datasets is below 25%, a common threshold for *ab initio *prediction [36].

We use sensitivity and specificity to evaluate the performance of SVMcon and CMAPpro. Sensitivity is the percentage of native contacts that are predicted to be contacts. Specificity is the percentage of predicted contacts that are present in the native structure. The contact threshold is set at 8 Å between Ca atoms. The sensitivity and specificity of a predictor depend also on the threshold used to separate 'contact' from 'non-contact' predictions. To compare SVMcon and CMAPpro fairly, we choose to evaluate them at their break-even point, where sensitivity is equal to specificity as in [37]. At the break-even point, the sensitivity and specificity of SVMcon is 27.1%, 4% higher than CMAPpro. Thus on the same benchmark dataset, SVMcon yields a sizable improvement.

We also compare the accuracy of SVMcon with the random uniform baseline algorithm consisting of random independent coin flips to decide whether each residue pair is in contact or not. Since the medium-and long-range contacts account for 2.8% of the total number of residue pairs with linear separation >= 6, the probability for the coin to produce a contact is set to 2.8%. As a result, the sensitivity and specificity of the random baseline algorithm is 2.8% at the break-even point. Thus SVMcon yields a nine-fold improvement over the random baseline.

Since the contact prediction accuracy varies significantly with individual proteins and their structure classes [29], we calculate the contact prediction specificity (or called accuracy) and sensitivity (or called coverage) for each test protein (Table 1). For each one, we select up to *L *(protein length) predicted contacts ranked by SVM scores because the total number of true contacts is approximately linear to the protein length [24]. The results show that in many cases (e.g. 1QJPA, 1DZOA, 1MAIA, 1LSRA, 1F4PA, 1MSCA, 1IG5A, 1ELRA, 1J75A), the prediction accuracy and coverage are > 30%.

However, for some proteins such as 1SKNP, the prediction accuracy is pretty low. We observe that the contact prediction accuracy is related to the the quality of multiple sequence alignment, the prediction accuracy of secondary structure, and the proportion of beta-sheets. Consistent with previous research [29,37], the contacts within beta-sheets in beta, alpha+beta, and alpha/beta proteins are predicted with higher accuracy than the contacts between an alpha helix and a beta strand or between alpha helices. We think that the strong restraints between beta-strands such as hydrogen-bonding probably contribute to the improved accuracy.

**Table 1:** Detailed Contact Prediction Results on 48 Test Proteins for Sequence Separation >= 6, 12, and 24 respectively.

Protein type	Len	Type	Separation >= 6		Separation >= 12		Separation >= 12	
			Acc(corr/pred)	Cov(corr/tot)	Acc(corr/pred)	Cov(corr/tot)	Acc(corr/pred)	Cov(corr/tot)
1IG5A	75	alpha	0.333 (25/75)	0.446 (25/56)	0.240 (18/75)	0.486 (18/37)	0.273 (9/33)	0.346 (9/26)
1HXIA	112	alpha	0.304 (34/112)	0.270 (34/126)	0.214 (24/112)	0.238 (24/101)	0.015 (1/67)	0.018 (1/55)
1SKNP	74	alpha	0.093 (4/43)	0.133 (4/30)	0.000 (0/18)	0.000 (0/24)	0.000 (0/6)	0.000 (0/20)
1ELRA	128	alpha	0.406 (52/128)	0.327 (52/159)	0.384 (33/86)	0.264 (33/125)	0.227 (5/22)	0.085 (5/59)
1E29A	135	alpha	0.289 (39/135)	0.193 (39/202)	0.111 (15/135	0.112 (15/134)	0.103 (7/68)	0.071 (7/99)
1CTJA	89	alpha	0.157 (14/89)	0.147 (14/95)	0.112 (10/89	0.204 (10/49)	0.090 (8/89)	0.190 (8/42)
1J75A	57	alpha	0.474 (27/57)	0.466 (27/58)	0.250 (7/28)	0.206 (7/34)	0.500 (1/2)	0.038 (1/26)
1ECAA	136	alpha	0.103 (14/136)	0.156 (14/90)	0.063 (5/79)	0.064 (5/78)	0.070 (3/43)	0.041 (3/74)
1FIOA	190	alpha	0.143 (19/133)	0.161 (19/118)	0.153 (11/72)	0.113 (11/97)	0.140 (8/57)	0.110 (8/73)
1C75A	71	alpha	0.282 (20/71)	0.211 (20/95)	0.099 (7/71)	0.127 (7/55)	0.087 (4/46)	0.089 (4/45)
1HCRA	52	alpha	0.058 (3/52)	0.231 (3/13)	0.056 (1/18)	0.167 (1/6)	0.000 (0/0)	0.000 (0/3)
1QJPA	137	beta	0.518 (71/137)	0.183 (71/389)	0.489 (67/137)	0.215 (67/312)	0.350(48/137)	0.300 (48/160)
1D2SA	170	beta	0.482 (82/170)	0.180 (82/455)	0.341 (58/170)	0.150 (58/386)	0.165 (28/170)	0.096 (28/293)
1CQYA	99	beta	0.182 (18/99)	0.080 (18/225)	0.172 (17/99)	0.094 (17/180)	0.273 (27/99)	0.197 (27/137)
1BMGA	98	beta	0.398 (39/98)	0.177 (39/220)	0.398 (39/98)	0.211 (39/185)	0.429 (42/98)	0.323 (42/130)
1MAIA	119	beta	0.538 (64/119)	0.298 (64/215)	0.361 (43/119)	0.250 (43/172)	0.034 (4/119)	0.048 (4/83)
1AMXA	150	beta	0.387 (58/150)	0.162 (58/357)	0.300 (45/150)	0.148 (45/304)	0.220 (33/150)	0.141 (33/234)
1G3PA	192	beta	0.042 (8/192)	0.019 (8/420)	0.042 (8/192	0.023 (8/342)	0.036 (7/192)	0.026 (7/273)
1RSYA	135	beta	0.578 (78/135)	0.259 (78/301)	0.459 (62/135)	0.240 (62/258)	0.230 (31/135)	0.177 (31/175)
1WHIA	122	beta	0.492 (60/122)	0.201 (60/298)	0.459 (56/122	0.226 (56/248)	0.295 (36/122)	0.303 (36/119)
1HE7A	107	beta	0.280 (30/107)	0.183 (30/164)	0.327 (35/107)	0.254 (35/138)	0.346 (37/107)	0.394 (37/94)
1MWPA	96	a+b	0.365 (35/96)	0.178 (35/197)	0.385 (37/96)	0.236 (37/157)	0.292 (28/96)	0.311 (28/90)
1QGVA	130	a+b	0.338 (44/130)	0.198 (44/222)	0.338 (44/130)	0.221 (44/199)	0.385 (50/130)	0.279 (50/179)
1DBUA	152	a+b	0.434 (66/152)	0.208 (66/317)	0.276 (42/152)	0.162 (42/260)	0.151 (23/152)	0.111 (23/207)
1XERA	103	a+b	0.466 (48/103)	0.219 (48/219)	0.330 (34/103)	0.214 (34/159)	0.204 (21/103)	0.193 (21/109)
1JSFA	130	a+b	0.500 (65/130)	0.316 (65/206)	0.385 (50/130)	0.345 (50/145)	0.154 (20/130)	0.235 (20/85)
1DZOA	120	a+b	0.608 (73/120)	0.330 (73/221)	0.500 (60/120)	0.351 (60/171)	0.083 (10/120)	0.139 (10/72)
1GRJA	151	a+b	0.318 (48/151)	0.209 (48/230)	0.225 (34/151)	0.186 (34/183)	0.066 (10/151)	0.084 (10/119)
1MSCA	129	a+b	0.620 (80/129)	0.421 (80/190)	0.512 (66/129)	0.524 (66/126)	0.225 (29/129)	0.644 (29/45)
1CEWI	108	a+b	0.528 (57/108)	0.300 (57/190)	0.454 (49/108)	0.310 (49/158)	0.278 (30/108)	0.316 (30/95)
1VHHA	157	a+b	0.414 (65/157)	0.206 (65/316)	0.338 (53/157	0.201 (53/264)	0.223 (35/157)	0.174 (35/201)
1BUOA	121	a+b	0.298 (36/121)	0.300 (36/120)	0.207 (25/121)	0.291 (25/86)	0.140 (17/121)	0.309 (17/55)
1G2RA	94	a+b	0.340 (32/94)	0.254 (32/126)	0.309 (29/94)	0.309 (29/94)	0.234 (22/94)	0.400 (22/55)
1E9MA	106	a+b	0.387 (41/106)	0.186 (41/220)	0.358 (38/106)	0.200 (38/190)	0.311 (33/106)	0.210 (33/157)
1E87A	117	a+b	0.470 (55/117)	0.239 (55/230)	0.299 (35/117)	0.193 (35/181)	0.291 (34/117)	0.227 (34/150)
1H9OA	108	a+b	0.630 (68/108)	0.354 (68/192)	0.352 (38/108)	0.299 (38/127)	0.148 (16/108)	0.302 (16/53)
1IDOA	184	a/b	0.402 (74/184)	0.223 (74/332)	0.402 (74/184)	0.250 (74/296)	0.402 (74/184)	0.277 (74/267)
1CHDA	198	a/b	0.429 (85/198)	0.175 (85/487)	0.384 (76/198)	0.170 (76/447)	0.338 (67/198)	0.181 (67/370)
1FUEA	163	a/b	0.374 (61/163)	0.185 (61/330)	0.374 (61/163)	0.206 (61/296)	0.399 (65/163)	0.251 (65/259)
1CXQA	143	a/b	0.448 (64/143)	0.303 (64/211)	0.350 (50/143)	0.276 (50/181)	0.091 (13/143)	0.115 (13/113)
1F4PA	147	a/b	0.442 (65/147)	0.222 (65/293)	0.361 (53/147)	0.205 (53/258)	0.354 (52/147)	0.223 (52/233)
1ES8A	196	a/b	0.240 (47/196)	0.130 (47/361)	0.153 (30/196)	0.100 (30/300)	0.189 (37/196)	0.160 (37/231)
1DMGA	172	a/b	0.302 (52/172)	0.176 (52/296)	0.273 (47/172)	0.175 (47/269)	0.192 (33/172)	0.155 (33/213)
1A1HA	85	small	0.424 (36/85)	0.424 (36/85)	0.129 (11/85)	0.262 (11/42)	0.000 (0/85)	0.000 (0/0)
9WGAB	171	small	0.415 (71/171)	0.188 (71/378)	0.357 (61/171)	0.268 (61/228)	0.041 (7/171)	0.175 (7/40)
2MADL	124	small	0.274 (34/124)	0.106 (34/321)	0.226 (28/124)	0.106 (28/263)	0.218 (27/124)	0.116 (27/232)
1EJGA	46	small	0.261 (12/46)	0.203 (12/59)	0.419 (13/31	0.271 (13/48)	0.458 (11/24	0.306 (11/36)
1AAOA	113	coil-coil	0.221 (25/113)	0.397 (25/63)	0.031 (3/97)	0.158 (3/19)	0.000 (0/35)	0.000 (0/0)

Column 1 lists the protein name (PDB code + chain id). The chain id of a single-chain protein is denoted by "A" instead of "-". Column 2 lists chain lengths, ranging from 46 to 198. Column 3 lists the SCOP structure class, alpha, beta, a+b, a/b, small, and coil-coil represent six SCOP protein classess (all alpha helix, all beta sheet, alpha helix + beta sheet, alpha helix alternating beta sheet, small protein, and coil-coil), respectively. Columns 4 and 5 report the prediction accuracy (specificity) and coverage (sensitivity) for each protein. Accuracy is the number of correct predictions divided by the total number of predictions. Coverage is the number of correct predictions divided by the total number of true contacts. The raw number of correct preditions, all predictions, and true contacts are also reported in the brackets.

**Figure 1:**
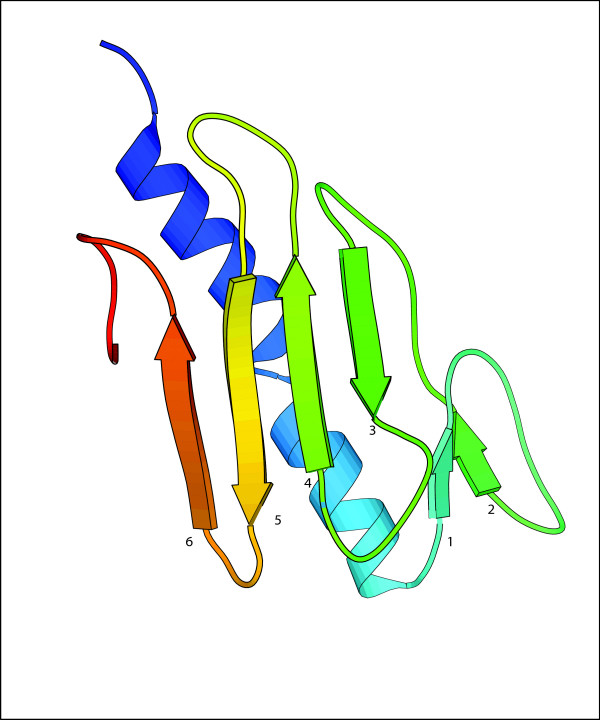
**3D Structure of Protein 1DZOA**. Protein 1DZOA is an a+b protein. It consists of two alpha helices and two beta sheets. Beta strands 1 and 2 form a parallel beta sheet. Beta strands 3, 4, 5, 6 form an anti-parallel beta sheet. Most non-local contacts involve the pairing interations between beta strands and the packing interactions between helices and beta sheets. (Figure rendered using Molscript [63]).

**Figure 2:**
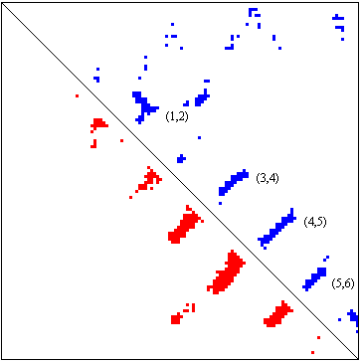
**Predicted and True Contact Maps of 1DZOA**. The upper triangle shows the true contacts of protein 1DZOA. The lower triangle shows the predicted contacts of protein 1DZOA. 2L (240) top ranked contacts are selected. The key contacts within anti-parallel strand pairs (3,4), (4,5), and (5,6) are recalled. A few contacts within the parallel strand pair (1,2) are also predicted correctly. However, very long range contacts between alpha helices and beta sheets are not predicted. And there are some false positives. It is interesting to see that most false positives are close to the true contacts. Thus, they may not be very harmful when being used as distance restraints to reconstruct protein 3D structure.

Figures 1 and 2 show the native 3D structure and the predicted contact map of a good example (protein 1DZOA), respectively. In this case, *2L *(240) predicted contacts with sequence separation >= 6 are selected. The contact prediction accuracy and coverage are 39.2% and 42.5%, respectively. It is shown that the predicted contact clusters (Figure 2) recall most native beta-sheet pairing patterns of the protein (Figure 1). And it is interesting to see most false positive contacts are also clustered around the true contacts. Thus, these noise may not be very harmful during the process of reconstructing protein structure from the contacts.

Furthermore, to investigate the relationship between the SVM contact map predictions and the structure classes, we compute the average accuracy and coverage of contact predictions in the six SCOP [38] structure classes (Table 2). The contact prediction accuracy of proteins having beta-sheets (alpha+beta, alpha/beta, beta) is higher than that of alpha helical proteins, which is consistent with previous observations [29]. According to Table 2, the average coverage is about 20% and the accuracy ranges from 21 to 37%. This level of accuracy is probably good enough (or at least helpful) for constructing an *ab initio *low-resolution structure, since previous experiments show that only *L*/5 or even less residues contacts are required to reconstruct a low resolution structure for a small protein [5,8,39-42], taking into account the inherent physical restraints of protein structures. However, the challenge is to develop algorithms to reconstruct a protein structure from a noisy predicted contact map, where contact restraints are much less reliable than the experimental contacts determined by NMR techniques.

**Table 2:** Contact Prediction Results of Proteins in the Six SCOP Structure Classes.

SCOP Class	Num	Separation >= 6		Separation >= 12		Separation >= 24	
		Accuracy	Coverage	Accuracy	Coverage	Accuracy	Coverage
alpha	11	0.24	0.24	0.17	0.18	0.11	0.09
beta	10	0.38	0.17	0.32	0.17	0.22	0.17
a+b	15	0.45	0.25	0.35	0.25	0.21	0.23
a/b	7	0.37	0.19	0.33	0.19	0.28	0.20
small	4	0.36	0.18	0.28	0.19	0.11	0.15
coil-coil	1	0.22	0.40	0.03	0.16	0.00	--

average	48	0.37	0.21	0.30	0.20	0.21	0.19

Column 1 lists six structure classes. Column 2 lists the number of proteins in each class. Other columns reports the accuracy and coverage of contact predictions in each category. The statistics is computed for sequence separation >= 6, 12, and 24, respectively. The last row reports the average performance on all 48 proteins. The accuracy of a+b and a/b is slightly higher than that of beta proteins, which is in turn higher than that of alpha proteins. The performance on small proteins (mostly alpha helical) lies between proteins containing beta-sheets (a+b, a/b, and beta) and alpha helical proteins. There is only one coil-coil protein, which does not have native contacts with sequence separation >= 24.

### Comparison with seven other Predictors during CASP7

SVMcon participated in the CASP7 experiment in 2006 and was evaluated along with seven other contact map predictors. The CASP7 evaluation procedure focuses on inter-residue contact predictions with linear sequence separation >= 12 and >= 24 respectively [15]. Up to *L*/5 of the top predicted contacts were assessed, where *L *is the length of the target protein. These evaluation metrics are also similar to those used in the past editions of the Critical Assessment of Fully Automated Structure Prediction Methods [43-45] and in the EVA contact evaluation server [46]. We use the similar procedure to compute accuracy (specificity) and coverage (sensitivity) for all server predictors.

We compare SVMcon with the other contact map predictors on the 13 out of 15 CASP7 *de novo *domains whose structures have been released. The contact map predictors participating in CASP7 include SVMcon, BETApro [37], SAM-T06 [47], PROFcon [32], GajdaPairings, Distill [34,48], Possum [19], and GPCPRED [29]. Their contact predictions were downloaded from the CASP7 website.

Table 3 reports the performance of the eight automated contact map predictors in the CASP7 experiment. The accuracy and coverage of SVMcon at a sequence separation threshold of 12 are 27.7% and 4.7% respectively, corresponding to the second best ranking behind our other predictor BETApro. The accuracy and coverage of SVMcon at a sequence separation threshold of 24 are 13.1% and 2.8% respectively, overall slightly behind SAM-T06 and BETApro. Its coverage at a sequence separation threshold of 24 is higher than Distill, Possum, GPCPRED, and GadjaPairings. Since PROFcon only made predictions for 11 out of 13 domains, its performance can not be directly compared with other methods. Here we include its results for completeness.

**Table 3:** CASP7 Results of Inter-Residue Contact Predictions of Eight Predictors.

	Separation >= 12		Separation >= 24	
Method	Acuracy (%)	Coverage (%)	Accuracy (%)	Coverage (%)
SVMcon	27.7	4.7	13.1	2.8
BETApro	35.4	5.1	19.7	3.2
SAM-T06	20.7	3.5	18.5	3.9
Distill	26.4	2.9	13.7	1.4
Possum	15.0	2.3	21.4	2.6
PROFcon	12.1	2.0	8.1	1.6
GPCPRED	12.2	2.1	10.5	2.0
GajdaPairings	9.8	1.5	10.4	1.9

The eight contact map predictors are evaluated on the 13 *de novo *domains of CASP7. The 13 domains include (T0296, T0300, T0307, T0309, T0314, T0316 domain 2, T0319, T0347 domain 2, T0350, T0353, T0361, T0356 domain 1, T0356 domain 3). The experimental structures of the targets and the domain classification can be found at the CASP7 web site (). The accuracy and coverage of contact predictions are evaluated at sequence separation >= 12 and >= 24, respectively. It is worth noting that PROFcon only made predictions for 11 out of 13 domains. Thus its performance can not be directly compared with other methods. Here we includes its results for completeness.

Another caveat is that the evaluation dataset and scheme we used may be slightly different from the official CASP7 evaluation. Thus, here we only try to evaluate the current state of the art of contact predictors instead of ranking them. For the offical contact evaluation scheme and results, readers are advised to check the assessment paper of the CASP7 contact predictions published in the upcoming supplement issue of the journal Proteins.

Overall, these results on the CASP7 dataset show that the accuracy and coverage of protein contact prediction are still low. However, these results are an important step towards reaching the milestone of an accuracy level of about 30%, required for deriving moderately accurate (low resolution) 3D protein structures from scratch [5,8,39-42] (Also, Dr. Yang Zhang, personal communication at the CASP7 conference). In particular, these predictors tend to predict different correct contacts. Thus, a consensus combination of contact map predictors may yield more accurate contact map predictions, which in turn could significantly improve 3D structure reconstruction. Since the stakes of contact map prediction are high, a community-wide effort for improving contact map prediction should be worthwhile (Dr. Burkhard Rost's lecture slides at Columbia University).

It is also worth pointing out that the CASP7 *de novo *dataset is too small to reliably estimate the accuracy of the predictors. So one should not over-interpret these results. Indeed, when we use a larger CASP *de novo *dataset of 24 domains classified by Dr. Dylan Chivian from Dr. David Baker's group to evaluate the predictors (results not shown), the accuracy of SVMcon and BETApro are very close for both sequence separations >= 12 and 24, and both remain among the top predictors.

## Conclusion

We have described a new contact map predictor (SVMcon) that uses support vector machines to integrate a large number of useful information including profiles, secondary structure, solvent accessibility, contact potentials, residue types, segment window information [24,32], and protein-level information [32]. The method yields a 4% improvement over the state-of-the art contact map predictor CMAPpro. In the blind CASP7 experiment, SVMcon is ranked as one of the top contact predictors. The method represents an effort toward a good 2D structure prediction. It can be used to improve *ab initio *structure prediction [4] and analogous fold recognition [2,3]. The web server, software, and source code are available at the SVMcon website [49].

## Methods

### Data Sets

In the comparison with CMAPpro [28,35], we use the same training and testing datasets. The datasets are redundancy reduced. The pairwise sequence identity of any two sequences is less than 25%. The training and testing datasets contain 485 sequences and 48 sequences respectively.

We use PSI-BLAST to search each sequence against the NCBI non-redundant database and generate a multiple sequence alignment. The number of PSI-BLAST iterations is set to 3. The e-value for selecting a sequence is set to 0.001. The e-value for including a sequence into the construction of a profile is set to 10^-10^. Multiple sequence alignments are converted into profiles, where each position is associated with a vector denoting the probability of each residue type.

We extract only medium- and long- range residue pairs with sequence separation >= 6 as in [32], which are not captured by local secondary structures. We use a 8 Å distance threshold between Ca atoms to classify residue pairs into two categories: contact (positive, < 8 Å) or non-contact (negative, >= 8 Å). Since the majority of residue pairs are negative examples, to balance the number of positive and negative examples in the training set we randomly sample and retain only 5% of the negative examples while keeping all positive examples. In total, there are 220,994 negative examples and 94,110 positive examples in the training data set. We keep all negative and positive examples in the test data set. The test data set has 10,498 positive examples and 367,299 negative examples.

### Input Features

We extract five categories of features for each pair of residues at positions *i *and *j *to evaluate their contact likelihood. In addition to the new features (e.g. pairwise information features), the choice of most features combines ideas from our previous contact map predictors, disulfide bond predictors [33,50], and beta sheet topology predictors [37], and from the PROFcon [32], the best predictor in CASP6.

#### Local window feature

We extract local features using a 9-residue window centered at each residue in each residue pair. For each position in the window, we use 21 inputs for the probabilities of the 20 amino acids plus gap, computed from multiple sequence alignments, 3 binary inputs for secondary structure (helix: 100, sheet: 010, coil: 001), 2 binary inputs for relative solvent accessibility (exposed: 10, buried: 01) at 25% threshold, one input for the entropy (- ∑_*k *_*p*_*k *_*logp*_*k*_) as a measure of local conservation. Here *p*_*k *_is the probability of occurrence of the *k*-th residue (or gap) at the position under consideration. Secondary structure and relative solvent accessibility are predicted using the SSpro and ACCpro programs in the SCRATCH suite [27,35,51]. Thus the two local windows produce 2 × 9 × 27 features.

#### Pairwise information features

For each pair of positions (*i*, *j*) in a multiple sequence alignment, we compute the following features. One input corresponds to the mutual information of the profiles of the two positions (∑_*kl *_*p*_*kl *_*log *(*p*_*kl*_/(*p*_*k*_*p*_*l*_)), where *p*_*kl *_is the empirical probability of residues (or gap) *k *and *l *appearing at the two positions *i *and *j *simultaneously. Two other pairwise inputs are computed using the cosine (x⋅y|x||y|
 MathType@MTEF@5@5@+=feaafiart1ev1aaatCvAUfKttLearuWrP9MDH5MBPbIqV92AaeXatLxBI9gBaebbnrfifHhDYfgasaacH8akY=wiFfYdH8Gipec8Eeeu0xXdbba9frFj0=OqFfea0dXdd9vqai=hGuQ8kuc9pgc9s8qqaq=dirpe0xb9q8qiLsFr0=vr0=vr0dc8meaabaqaciaacaGaaeqabaqabeGadaaakeaadaWcaaqaaiabdIha4jabgwSixlabdMha5bqaamaaemaabaGaemiEaGhacaGLhWUaayjcSdWaaqWaaeaacqWG5bqEaiaawEa7caGLiWoaaaaaaa@3B32@) and correlation (∑i(xi−x¯)(yi−y¯)∑i(xi−x¯)2∑i(yi−y¯)2
 MathType@MTEF@5@5@+=feaafiart1ev1aaatCvAUfKttLearuWrP9MDH5MBPbIqV92AaeXatLxBI9gBaebbnrfifHhDYfgasaacH8akY=wiFfYdH8Gipec8Eeeu0xXdbba9frFj0=OqFfea0dXdd9vqai=hGuQ8kuc9pgc9s8qqaq=dirpe0xb9q8qiLsFr0=vr0=vr0dc8meaabaqaciaacaGaaeqabaqabeGadaaakeaadaWcaaqaamaaqababaGaeiikaGIaemiEaG3aaSbaaSqaaiabdMgaPbqabaGccqGHsislcuWG4baEgaqeaiabcMcaPiabcIcaOiabdMha5naaBaaaleaacqWGPbqAaeqaaOGaeyOeI0IafmyEaKNbaebacqGGPaqkaSqaaiabdMgaPbqab0GaeyyeIuoaaOqaamaakaaabaWaaabeaeaacqGGOaakcqWG4baEdaWgaaWcbaGaemyAaKgabeaakiabgkHiTiqbdIha4zaaraGaeiykaKYaaWbaaSqabeaacqaIYaGmaaGcdaaeqaqaaiabcIcaOiabdMha5naaBaaaleaacqWGPbqAaeqaaOGaeyOeI0IafmyEaKNbaebacqGGPaqkdaahaaWcbeqaaiabikdaYaaaaeaacqWGPbqAaeqaniabggHiLdaaleaacqWGPbqAaeqaniabggHiLdaaleqaaaaaaaa@55C8@) measures on the profiles at positions *i *and *j*. Thus some information about correlated mutations is used in the inputs. We also use three inputs to represent Levitt's contact potential [52], Jernigan's pairwise potential [53], and Braun's pairwise potential [54] for the residue pairs in the target sequence.

#### Residue type features

We classify residues into four categories: non-polar (G, A, V, L, I, P, M, F, W), polar (S, T, N, Q, C, Y), acidic (D, E), basic (K, R, H). These four residue types induce 10 different combinations: non-polar/non-nopolar, non-polar/polar, non-polar/acidic, non-polar/basic, polar/polar, polar/acidic, polar/basic, acidic/acidic, acidic/basic, and basic/basic. We use 10 binary inputs to encode the type of a residue pair.

#### Central segment window feature

Central segment window corresponding to a window centered at position ⌊(*i *+ *j*)/2)⌋ has been shown to be useful for predicting whether the residues at position *i *and *j *are in contact or not [24,32]. We use a central segment window of size 5. For each position in the window, we use the same 27 features as the local window features. So the total number of features for the central window is 5 × 27. We also compute the amino acid composition (21 features), secondary structure composition (3 features), relative solvent accessibility composition (2 features) in the central segment window. The sequence separation (|*i *- *j *+ 1|) for residue pair (*i*, *j*) are classified into one of 16 length bins (< 6, 6, 7, 8, 9, 10, 11, 12, 13, 14, < 19, < 24, <= 29, <= 39, <= 49, >= 50) using a binary vector of length 16, as in [32].

#### Protein information features

We also compute the global amino acid composition (21 features), secondary structure composition (3 features), and relative solvent accessibility composition (2 features) of the target sequence. In addition, we classify sequence lengths into four bins (<= 50, <= 100, <= 150, and > 150) using a binary vector of length 4, as in [32].

The detailed methods of generating features are described in the additional files [see Additional file 1, 2, 3].

### Feature Selection

Feature selection is useful to improve the performance of machine learning methods, particularly when there is a large number of features as in this study. However, since there are more than 310,000 training data points, it takes about 12 days to conduct a round of training and testing on a Pentium-IV computer. Thus a thorough feature selection is currently not feasible. So we tried only to remove some features (pairwise profile correlation, pairwise mutual information, residue type, and protein information features) once a time to test how they affect the prediction accuracy. We find that removing these features slightly improve the accuracy by about 0.2%. However, it is not clear if the improvement is due to the random variation or due to the removal of the features. But at least, these features are not essential or being compensated by other similar features. Thus, a more thorough feature selection should be conducted to improve the performance when more computing power is available.

### SVM Learning

For an input feature vector associated with a pair of residues, we use Support Vector Machines (SVMs) to predict if the two residues are in contact (positive) or not (negative). SVMs provide a non-linear classifier model by non-linearly mapping the input vectors into a feature space and using linear methods for classification in the feature space [55-58]. Thus SVMs, and more generally kernel methods, attempt to combine the advantages of both linear and nonlinear methods by first embedding the data into a feature space equipped with a dot product and then using linear methods in the feature space to perform classification or regression tasks based on the Gram matrix of dot products between data points. A key property of kernel methods is that the embedding does not need to be given in explicit form, the Gram matrix of dot products *K *(*x*, *y*) = *φ *(*x*)·*φ *(*y*) between data points is all is needed to proceed with classification or regression. Here *x *and *y *are input data points, *φ *is the mapping from input space to feature space, and *K *is the kernel or similarity measure between the original data points. Given a set of training data points *S *= *S*^+ ^∪ *S*^-^, where *S*^+ ^(resp. *S*^-^) represent the positive (resp. negative) examples, using the theory of structural risk minimization [55-58], SVMs learn a classification function *f *(*x*) in the form of

f(x)=∑xi∈S+αiK(x,xi)−∑xi∈S−αiK(x,xi)+b
 MathType@MTEF@5@5@+=feaafiart1ev1aaatCvAUfKttLearuWrP9MDH5MBPbIqV92AaeXatLxBI9gBaebbnrfifHhDYfgasaacH8akY=wiFfYdH8Gipec8Eeeu0xXdbba9frFj0=OqFfea0dXdd9vqai=hGuQ8kuc9pgc9s8qqaq=dirpe0xb9q8qiLsFr0=vr0=vr0dc8meaabaqaciaacaGaaeqabaqabeGadaaakeaacqWGMbGzcqGGOaakcqWG4baEcqGGPaqkcqGH9aqpdaaeqbqaaGGaciab=f7aHnaaBaaaleaacqWGPbqAaeqaaOGaem4saSKaeiikaGIaemiEaGNaeiilaWIaemiEaG3aaSbaaSqaaiabdMgaPbqabaGccqGGPaqkaSqaaiabdIha4naaBaaameaacqWGPbqAaeqaaSGaeyicI4Saem4uam1aaWbaaWqabeaacqGHRaWkaaaaleqaniabggHiLdGccqGHsisldaaeqbqaaiab=f7aHnaaBaaaleaacqWGPbqAaeqaaOGaem4saSKaeiikaGIaemiEaGNaeiilaWIaemiEaG3aaSbaaSqaaiabdMgaPbqabaGccqGGPaqkaSqaaiabdIha4naaBaaameaacqWGPbqAaeqaaSGaeyicI4Saem4uam1aaWbaaWqabeaacqGHsislaaaaleqaniabggHiLdGccqGHRaWkcqWGIbGyaaa@5E31@

where *α*_*i *_are non-negative weights assigned to the training data point *x*_*i *_during training by minimizing a quadratic objective function and *b *is the bias. Thus the function *f *(*x*) can be viewed as a weighted linear combination of similarities between training data points *x*_*i *_and the target data point *x*. Only data points with strictly positive weight *α *in the training dataset affect the final solution. The corresponding data points *x*_*i *_are called the support vectors. For contact map prediction, a new data point *x *is predicted to be positive or negative by taking the sign of *f *(*x*).

We use SVM-light [59-61] to implement SVM classification on our data. We experimented with several common kernels including linear kernels, Gaussian radial basis kernels (RBF), polynomial kernels, and sigmoidal kernels. In our experience, on this data the RBF kernel *K *(*x*, *y*) = e−γ‖x−y‖2
 MathType@MTEF@5@5@+=feaafiart1ev1aaatCvAUfKttLearuWrP9MDH5MBPbIqV92AaeXatLxBI9gBaebbnrfifHhDYfgasaacH8akY=wiFfYdH8Gipec8Eeeu0xXdbba9frFj0=OqFfea0dXdd9vqai=hGuQ8kuc9pgc9s8qqaq=dirpe0xb9q8qiLsFr0=vr0=vr0dc8meaabaqaciaacaGaaeqabaqabeGadaaakeaacqWGLbqzdaahaaWcbeqaaiabgkHiTGGaciab=n7aNnaafmaabaGaemiEaGNaeyOeI0IaemyEaKhacaGLjWUaayPcSdWaaWbaaWqabeaacqaIYaGmaaaaaaaa@38EF@ (or e−‖x−y‖2σ2
 MathType@MTEF@5@5@+=feaafiart1ev1aaatCvAUfKttLearuWrP9MDH5MBPbIqV92AaeXatLxBI9gBaebbnrfifHhDYfgasaacH8akY=wiFfYdH8Gipec8Eeeu0xXdbba9frFj0=OqFfea0dXdd9vqai=hGuQ8kuc9pgc9s8qqaq=dirpe0xb9q8qiLsFr0=vr0=vr0dc8meaabaqaciaacaGaaeqabaqabeGadaaakeaacqWGLbqzdaahaaWcbeqaaiabgkHiTmaalaaabaWaauWaaeaacqWG4baEcqGHsislcqWG5bqEaiaawMa7caGLkWoadaahaaadbeqaaiabikdaYaaaaSqaaGGaciab=n8aZnaaCaaameqabaGaeGOmaidaaaaaaaaaaa@3A46@) works the best. Using the RBF kernel, *f *(*x*) is actually a weighted sum of Gaussians centered on the support vectors. Almost any separating boundary or regression function can be obtained with such a kernel [62], thus it is important to tune the SVM parameters carefully in order to achieve good generalization performance and avoid overfitting.

We only adjust the width parameter *γ *of the RBF kernel, leaving all other parameters to their default value. *γ *is the inverse of the variance (*σ*^2^) of the RBF and controls the width of the Gaussian functions centered on the support vectors. The bigger is *γ*, the more peaked are the Gaussians, and the more complex are the resulting decision boundaries [62]. After experimenting with several values of *γ*, we selected *γ *= 0.025.

## Authors' contributions

JC designed the features, implemented the algorithm, and carried out the experiment. JC and PB authored the manuscript. Both authors approved the manuscript.

## Supplementary Material

Additional file 1 The main Perl script to predict a contact map. It is a text file that can be viewed by any text viewer/editor. Click here for file

Additional file 2 The Perl script to generate input features for support vector machine. It is a text file that can be viewed by any text viewer/editor. Click here for file

Additional file 3 The Perl script to compute pairwise contact potentials. It is a text file that can be viewed by any text viewer/editor. Click here for file
